# Small molecule biomarker discovery: Proposed workflow for LC-MS-based clinical research projects

**DOI:** 10.1016/j.jmsacl.2023.02.003

**Published:** 2023-02-17

**Authors:** S. Rischke, L. Hahnefeld, B. Burla, F. Behrens, R. Gurke, T.J. Garrett

**Affiliations:** a pharmazentrum frankfurt/ZAFES, Institute of Clinical Pharmacology, Johann Wolfgang Goethe University, Theodor Stern-Kai 7, 60590 Frankfurt am Main, Germany; b Fraunhofer Institute for Translational Medicine and Pharmacology ITMP, and Fraunhofer Cluster of Excellence for Immune Mediated Diseases CIMD, Theodor-Stern-Kai 7, 60596 Frankfurt am Main, Germany; c Singapore Lipidomics Incubator, Life Sciences Institute, National University of Singapore, Singapore, Singapore; d Division of Rheumatology, University Hospital Frankfurt, Theodor-Stern-Kai 7, 60596 Frankfurt am Main, Germany; e Department of Pathology, Immunology and Laboratory Medicine and Southeast Center for Integrated Metabolomics, University of Florida, Gainesville, FL 32611, USA

**Keywords:** HILIC, Hydrophilic interaction liquid chromatography, HRMS, High resolution mass spectrometry, LC-MS, Liquid chromatography – mass spectrometry, MRM, Multiple reaction monitoring, PCA, Principal component analysis, QA, Quality assurance, QC, Quality control, RF, Random Forest, RP, Reversed phase, SVA, Support vector machine, (U)HPLC (Ultra-), High pressure liquid chromatography, LC-MS-Based Clinical Research, Biomarker Discovery Study, Lipidomics, Metabolomics

## Abstract

Mass spectrometry focusing on small endogenous molecules has become an integral part of biomarker discovery in the pursuit of an in-depth understanding of the pathophysiology of various diseases, ultimately enabling the application of personalized medicine. While LC-MS methods allow researchers to gather vast amounts of data from hundreds or thousands of samples, the successful execution of a study as part of clinical research also requires knowledge transfer with clinicians, involvement of data scientists, and interactions with various stakeholders.

The initial planning phase of a clinical research project involves specifying the scope and design, and engaging relevant experts from different fields. Enrolling subjects and designing trials rely largely on the overall objective of the study and epidemiological considerations, while proper pre-analytical sample handling has immediate implications on the quality of analytical data. Subsequent LC-MS measurements may be conducted in a targeted, semi-targeted, or non-targeted manner, resulting in datasets of varying size and accuracy. Data processing further enhances the quality of data and is a prerequisite for in-silico analysis. Nowadays, the evaluation of such complex datasets relies on a mix of classical statistics and machine learning applications, in combination with other tools, such as pathway analysis and gene set enrichment. Finally, results must be validated before biomarkers can be used as prognostic or diagnostic decision-making tools. Throughout the study, quality control measures should be employed to enhance the reliability of data and increase confidence in the results.

The aim of this graphical review is to provide an overview of the steps to be taken when conducting an LC-MS-based clinical research project to search for small molecule biomarkers.

## Highlights

• LC-MS centered biomarker studies can be displayed in five overlapping phases.
• Interdisciplinary work is of utmost importance for successful project realization.
• Complexity of LC-MS based studies poses unique challenges that need to be tackled.
• Each phase of LC-MS based clinical research has significant impact on study results.
• Data quality must be monitored and can be enhanced by quality control measures.

## Graphical abstract

**Figure:**
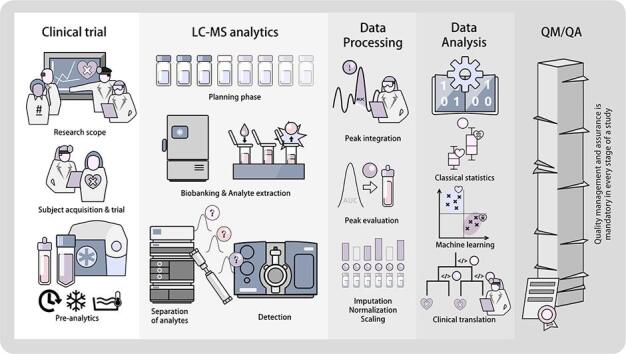

## Introduction

Liquid chromatography – mass spectrometry (LC-MS) aided biomarker discovery is a complex and extensive process that requires interdisciplinary expertise, horizontal knowledge transfer between clinicians, analysts, data scientists, and other stakeholders (e.g., patients), and detailed planning. This process can be divided into five interwoven phases, with quality control (QC) measures supervising the other phases and facilitating confidence in findings and reproducibility. The other stages include clinical trial (including sample collection and preprocessing), sample preparation (e.g., extraction), LC-MS analyses, data processing, and evaluation. The following graphical review outlines a general workflow for LC-MS-based clinical research projects that are intended to search for small molecule biomarkers or biomarker profiles.

## Study and trial design

**Fig. 1:**
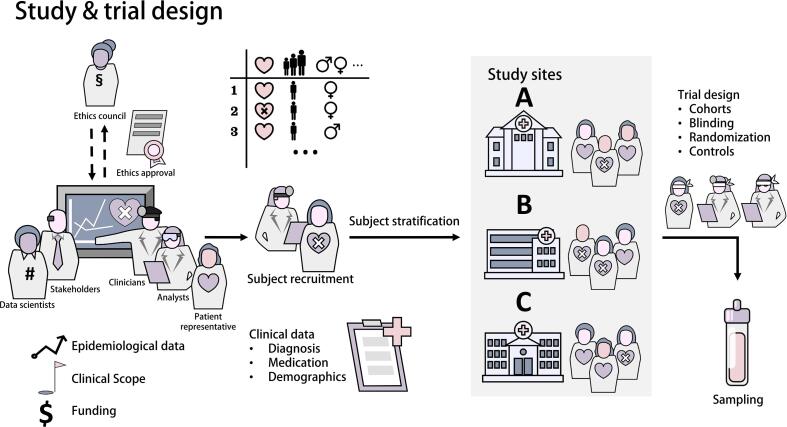
Before generating any sample, a clinical study for discovering biomarkers requires careful planning, including obtaining ethics approval, defining a research scope involving stakeholders such as clinicians, analysts, data scientists, investors, and patients, and obtaining sufficient funding. Subject recruitment and stratification are instrumental to subsequent evaluations. Documenting meta data such as clinical (e.g., BMI), individual (e.g., smoking habits), and demographic information (e.g., age) on study subjects is highly relevant for trial design, as well as subsequent data analyses. The trial design depends on a multitude of factors, including modes of randomization and blinding, as well as more practical considerations such as sampling plans and study sites.

Clinical research projects typically originate from observations of pre-clinical research or epidemiological data that suggest a need for a better understanding of pathophysiological processes, with the goal of improved diagnosis, prediction of progression, or selection of therapy in certain diseases [Fig. 1]. A plan is laid out based on literature, clinical relevance, and feasibility, including patient feedback, and funding is secured from either public organizations or industry [1]. It is important to discuss the implementation of potential biomarker screenings in terms of equipment, expertise, and capacity between end users and researchers early on. An ethics council must then evaluate the study protocol and give their approval. Before the first samples are collected, analytical scientists will develop or select the methods to be used in the project and specify the pre-analytical requirements for the compounds or compound classes of interest. Depending on the research goals, different trial designs should be evaluated, taking into account various strategies for cohort randomization (e.g., simple randomization, block randomization or stratified randomization) and blinding (e.g., double/triple blinding) [2], [3]. Meanwhile, subject recruitment and stratification will occur at the clinical site and samples will be taken and processed following predefined standard operating procedures [4]. Comprehensive subject data (e.g., age, gender, known diseases and medication) for later evaluation must be documented, while national and international standards for data privacy must be adhered to. Sampling protocols should carefully regulate sampling to control for pre-analytical variables. Pre-analytical factors are known to distort analyte levels and disrupt meaningful analysis, so emphasis should be put on sample handling that ensures sample integrity [5]. Sample documentation should include unique sample IDs, data on sample collection and the respective participant, using either unique sample-IDs or barcodes. Sample matrices, regulations in shipping biological specimens, sample stability, as well as distance between the clinical and analytical site must all be taken into account when planning the clinical research project [6], [7].

### Pre-analytics - sampling and sample preprocessing

**Fig. 2:**
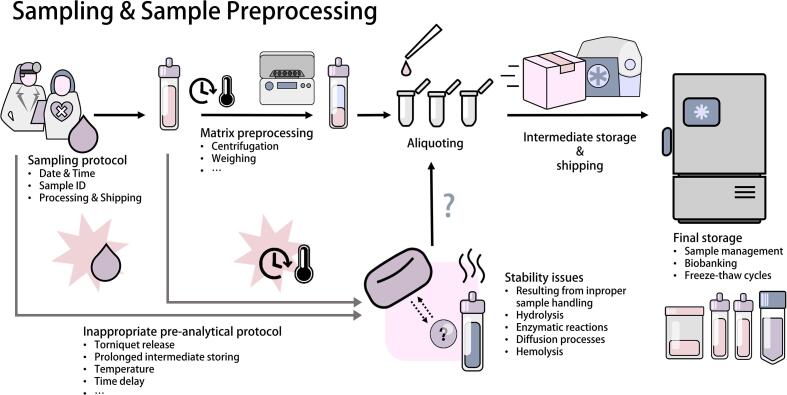
Sampling should follow a predefined protocol aligned with quality standards for the analytes of focus in the study. The pre-analytical phase in the life cycle of samples has the potential to significantly alter analyte concentrations. To ensure accurate results, well-defined procedures and relevant documentation on sample processing should be in place. Matrix preprocessing should be conducted at the sampling site, as whole blood cannot be stored or transported effectively without significant ex-vivo changes; it must be processed promptly into a plasma sample. When multiple measurements or technical replicates are planned for individual samples, aliquoting is required and should be conducted directly after sampling and before dry-ice shipping and final storage, usually at < -70 °C.

Sample preprocessing, including the generation of aliquots, should take place at the clinical site(s). Aliquot volumes should be selected appropriately to avoid unwanted freeze–thaw cycles. Further processing may also occur in the analytical laboratory before transferring samples to their final storage or starting the measurement. Special consideration should be placed on preserving sample stability in this phase as well. Analyte concentrations can be altered by simple chemical reactions, such as hydrolysis, enzymatic metabolism, or mass exchange between cells, or even the intrinsic instability of the compound of interest [8]. Therefore, the mode and temperature of intermediate storage, as well as the acceptable amount of processing delay should be predetermined. Sample handling steps for every sample need to be documented for tracking potential quality defects. For instance, in the case of anticoagulated whole blood samples, the time and temperature until centrifugation and separation of plasma, as well as centrifugation parameters and final storage temperature must be documented [Fig. 2] [9], [10], [11], [12]. In cases where the protocol of sample collection is not within the scope of the research (e.g., samples derived from biobanks), sample quality can be assessed retrospectively. Especially for blood specimens, various markers for sample quality defects have been described. For example, hemolysis in blood samples can be assessed by determining the hemolytic index [13], while other metabolomics biomarkers have been proposed for the identification of other pre-analytical risk factors, such as prolonged storage at room temperature [9], [14]. Without consensus on the best mode of detection of stability issues, documentation of sample collection remains an important tool for tracking potential quality defects. Aliquoting preprocessed matrices allows for obtaining standardized volumes from individual samples, as well as preparing technical replicates for different measurements and diluting matrices for the process of analyte extraction, which might be especially necessary for highly abundant compounds. The most important reason for generating small-volume aliquots is the prevention of additional freeze/thaw cycles, which can have strong effects on analyte concentration [15], [16]. Sample quality impairments, such as evaporation or oxidation, should be considered when generating very small aliquots. The effect of aliquot volume on the duration of thawing and subsequent stability implications should be considered as well [17]. As measurements are not necessarily performed immediately after sample preprocessing, biospecimens should be frozen at storage temperatures below −70 °C. When freeze–thaw cycles are kept to a minimum, deep freezing facilitates long-term storage and collection of sufficiently high sample numbers for performing LC-MS analytics [6]. Sample stability concerns related to the measurement should be pre-conceived during LC-MS method development and validation.

## Planning of LC-MS-analysis

LC-MS is able to quantify a wide variety of small molecules, including lipids or polar metabolites. The choice of methods depends largely on research requirements and the accessibility of compounds for analysis. Depending on whether the clinical study aims to answer highly specific or more general research questions, measurement can be conducted in targeted or non-targeted modes. Targeted LC-MS analysis involves the analysis of a predefined set of substances, often related by substance class or biological pathway, whereas non-targeted analysis records a broad set of mass traces that are identified and quantified later and usually result in comprehensive data sets [18]. Targeted analysis is often focused on low-concentration compounds, like lipid mediators, which require specific method optimization. LC-MS/MS is most commonly used for targeted analysis, due to its high sensitivity, selectivity, and robustness. Targeted analysis can also include absolute quantification, which requires the acquisition of reference substances and internal standards that are preferably isotopically labeled and allow for external calibration on an individual analyte level [19]. Absolute quantitative data allows for drawing conclusions regarding physiological reference intervals and simplifies data integration from multiple sites or studies. In the absence of substance-specific reference standards, relative quantification can be applied, either comparing the results to internal standards or to other samples (or reference materials) like a control group [20]. Non-targeted LC-HRMS analysis typically generates relative quantitative datasets of an extensive number of analytes, but focuses on species of higher abundance, due to a limited dynamic range [21]. Unlike targeted analysis, no predefinition of analytes is required for non-targeted approaches. To identify analytes, inferences are made from retention time and mass traces using previous experimental data and/or MS/MS libraries. To accelerate data evaluation, the analytes reviewed in a non-targeted analysis can be restricted post-acquisition to previously identified compounds, which is often referred to as semi-targeted analysis [22]. While the classical workflow of multiple reaction monitoring (MRM) allows for the adjustment of collision energy and de-clustering potential to optimize ion fragmentation and enhance sensitivity for a specific set of analytes [23], non-targeted approaches rely on a fixed combination of instrument parameters for a, generally, much broader analyte coverage [24]. Yet certain metabolites might still be elusive to the dynamic range of non-targeted setups. MS/MS approaches with a wide range of (scheduled) MRM transitions can serve as a compromise between the more reliable quantification of analytes provided by targeted analyses and the more extensive analyte coverage of non-targeted approaches [25].

**Fig. 3:**
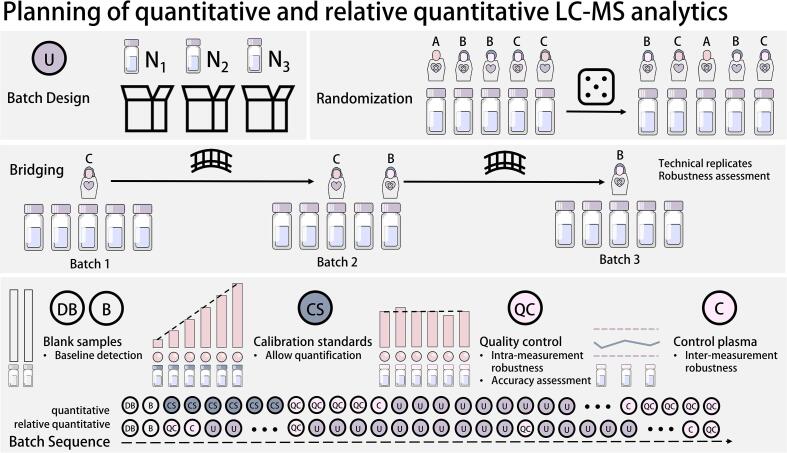
Meticulous planning of LC-MS measurements avoids pitfalls, like selection biases, and can be instrumental in quality control of the measurement. Initial batch design refers to dividing samples into different analytical runs. Different kinds of randomization can come into play, when aiming to mitigate the risk of individual samples being subjected to signal drift. While simple randomization is suitable for independent samples, a stratified cluster randomization might be more suitable for samples with nested dependence (e.g. time series data for individual subjects). Bridging refers to deliberately measuring technical replicates of samples, in order to connect batches by some proportion of shared measurements, allowing for comparability and assessment of robustness. The batch sequence (e.g., order of sample types) is subject to the mode of measurement. Depending on whether the measurement is absolute quantitative or relative quantitative, different combinations of blank samples, inter- and intra-measurement quality control samples, calibration standards and subject related unknown samples need to be considered. (Used abbreviations: U = “Sample of unknown concentration”; DB = “Double blank (without IS)”; B = “Blank (with IS)”; CS = “Calibration standard”; QC = “Quality control”; C = “Control plasma”).

Special care should be taken when planning the batch sequence for LC-MS analysis, regardless of whether it is targeted, semi-targeted, or non-targeted [Fig. 3]. Randomizing samples can mitigate the risk of bias due to signal drift [26]. Further considerations should be given to the placement of blank samples, calibration standards, QC samples, long-term control samples, and bridging samples (if applicable). Bridging samples are technical replicates of study samples between batches, which can be used to track the robustness of measurements over multiple batches. The batch size, among other factors, depends on the overall sample size, duration of measurement, and reasonable assumptions of sample stability [27].

The extraction of analytes from sample matrices refers to the isolation of compounds of interest while excluding or minimizing those that could interfere with the measurement. Analyte extraction can take different forms, ranging from protein precipitation, liquid–liquid extraction, to solid-phase extraction [21]. An internal or surrogate standard, which is distinguishable from the analyte yet behaves similarly in measurement (e.g., an isotopically labeled variant), is processed within the same sample. Since the amount of internal standards is held constant across samples, it is suitable for assessing analytical variability or matrix effects on a sample-by-sample basis [28]. In addition to samples, calibration standards (in the case of quantitative analysis), quality control (QC) samples, and long-term control samples are also prepared.

### Targeted LC-MS-analysis

**Fig. 4:**
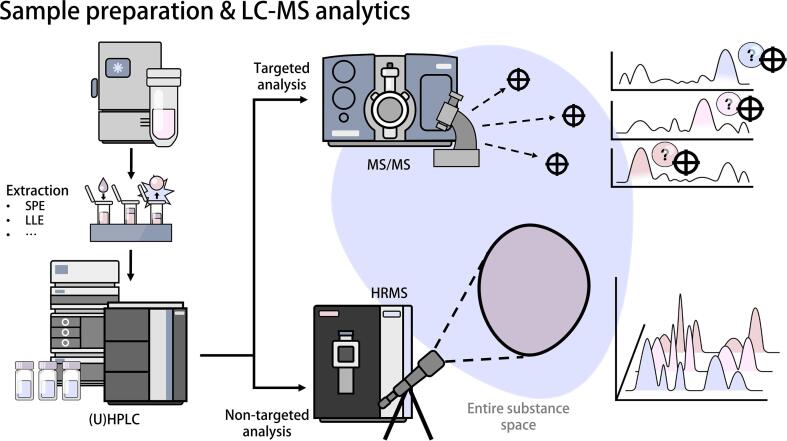
The process of LC-MS analysis begins with the extraction of analytes that are specific to the class. Each extraction method includes one or more purification steps. Internal or surrogate standards are added to the samples, which are processed alongside the analytes in question. This allows the signal of the analytes of interest to be related to the surrogate signal, as they are all subject to the same influences during extraction, such as matrix effects and other measurement-specific variations in sensitivity. In an often used setup, (U)HPLC is used to separate analytes, while mass spectrometry facilitates their detection. Targeted analysis using tandem mass spectrometry is limited to a predefined set of analytes, but, when combined with matching reference standards, it allows for absolute quantification. Non-targeted analysis using high resolution mass spectrometry has fewer restrictions on what analytes can be detected, allowing for simultaneous analysis of hundreds or thousands of features. However, without compound-specific reference standards, quantification is only relative. Semi-targeted analysis is a compromise between these two methods, usually focusing on hundreds of well-characterized analytes while granting broad insights at reduced processing times. As reference standards are limited in availability and economical viability, semi-targeted analysis is mostly only relative quantitative.

In the case of targeted and absolute quantitative LC-MS analytics, calibration standards and QC samples at different concentration levels are necessary [29]. Quantitative analysis is used to compare compound-specific calibration standards to an internal standard in order to enable the calculation of absolute analyte concentrations and to ensure confidence in compound identification. Retention time, fragmentation patterns, and peak shape of reference standards allow for drawing conclusions on analyte behavior. It is ideal to use isotopically labeled internal standards as analogues of the compound of interest. Calibration standards are derived by diluting and extracting reference substances in an analyte-free matrix to create a reference frame of concentrations (calibration curve), which can then be compared to samples of unknown concentration in order to calculate their relative concentrations [Fig. 3] [30]. Reference standard-based QC samples are processed with known concentrations spanning the range of measurable concentrations defined by the calibration curve, allowing for an assessment of accuracy and measurement system stability. In targeted analysis, these QC samples are usually placed at the beginning, intermittently at regular intervals, and at the end of the batch sequence so their accuracy and coefficient of variance can be used to determine measurement robustness and identify (and possibly correct for) drift effects within the batch [31]. Long-term control samples are similarly used as other QC samples, but concentration levels of technical replicates can be tracked in control charts over multiple measurements of the same or different studies in order to assess consistency of the LC-MS setup [32]. Thus, while regular QC samples serve as a control within the measurement, long-term control samples serve as a quality control method for the whole analytical process. Bridging samples are also technical replicates of individual samples that must be planned and prepared during sample aliquoting; one sample from each pair is measured in the batch following the first one. This is especially useful for assessing technical variations (i.e., batch effects) across multiple batches within the study. The selection for bridging samples should be done preemptively and randomized [33]. After completing the planning phase of the measuring sequence, extraction of analytes can begin. The measurement method, LC-column, and solvents should be specific to the chemical properties of the investigated analytes [Fig. 4]. Reversed phase (RP) columns are considered standard for most separation methods and are especially suitable for separation of lipophilic substances; however, polar metabolites may require Hydrophilic Interaction Liquid Chromatography (HILIC) phases in order to achieve suitable selectivity [35]. For targeted analysis, tandem mass spectrometers are often used due to their high selectivity (and wide linear range) and sensitivity, which is well-suited for analyzing a predefined set of analytes. While many targeted methods use absolute quantification, targeted screening methods also allow for analyzing hundreds of analytes without reference standards using relative quantification and a measurement setup similar to non-targeted methods [36].

### Semi- and Non-targeted LC-MS-analysis

In non-targeted analysis, features with unique combinations of mass-to-charge and retention time are aligned, annotated, and compared based on relative quantification. High resolution mass spectrometry is typically used to allow for compound annotation or identification based on exact mass, isotopic distribution, (relative) retention time, and by comparison of MS/MS fragmentation patterns to reference spectra [24]. This expands the range of potentially observable analytes compared to targeted analysis; however, it also increases the chance of false identification. Quantification is limited by the fact that many analytes are related to only few surrogate internal standards or relative sample concentrations are calculated in relation to all other samples. The signal intensity of compounds can vary based on carbon chain length, the number of double bonds, and structural differences [37]. The amount of relatively quantified features can easily reach into the thousands; therefore, internal standards cannot be selected on an individual analyte level. Absolute quantitative analysis with reference standard calibration is also not suitable for the same reasons. Long-term control samples can still be used for tracking system performance. QC samples, used for assessing measuring robustness within an analysis run, also do not require a range of concentrations; rather, they are replicates from a representative sample pool distributed over the batch at regular intervals (e.g., every 8 to 20 samples).

### Data integration and processing

**Fig. 5:**
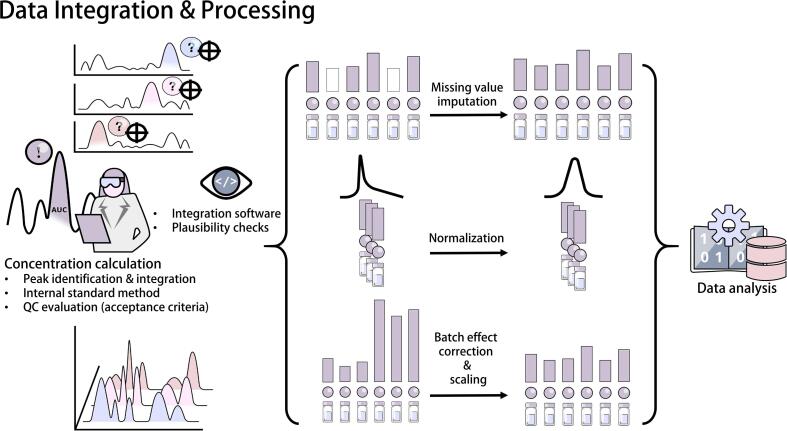
Peak identification depends on the mass traces and retention time of the respective analytes. Peak integration enables relative quantification. Relative quantification (non-targeted analysis) compares the peak areas of multiple analytes with one or multiple internal standards or a reference sample, and does not use a calibration curve of reference standards, which is necessary for targeted measurements. Further data processing might be needed after obtaining the results. Missing values must be imputed, outliers potentially removed, samples may need to be scaled for comparability, normalized to meet the assumptions of statistical models, and batch effects must be identified and corrected retrospectively.

Signal interpretation uses specialized software, either specific to the manufacturer or offered by third-party vendors, to identify and integrate signal peaks for each analyte and its respective internal standard. This is instrumental in calculating the concentration, as it relies on the area under the curve. The evaluation software usually automates significant proportions of peak annotation and integration when referencing a database of earlier measurements [38]. Exceptions include non-targeted analysis, where it might not be possible to find a suitable internal standard for all features, yet surrogates are still used to verify technical variation. Absolute concentrations are calculated using a calibration curve [30] and values that deviate from the line should be deemed indistinct and may be imputed in later steps. QC samples are utilized to evaluate measurement robustness, a key parameter in biomarker discovery [39]. These concentrations should align with other QC samples (e.g., pooled QC samples), and their accuracy and coefficient of variance serves as a measure of quality for the analysis run [40], [41]. Despite this, the resulting dataset might still be skewed and can be transformed further depending on its planned application [Fig. 5]. Missing values can be imputed, although the choice of method usually requires knowledge as to why the values are missing [42]. Erratically missing values may be imputed by more sophisticated methods like kNN or Random Forest Imputation [43], while values that fall below the lower limit of quantification can often be imputed with half of said limit, thus accounting for their approximate scale. Additionally, sample data can be normalized and scaled by various methods in order to remove systematic and statistical biases. Transformations towards normal distribution of measurements can be performed to satisfy the assumptions of statistical and machine learning models [34]. Normalization techniques include approaches of general applicability that aim for comparability of samples (e.g., Probabilistic Quotient Normalization [44] or Quantile Normalization [45]), as well as techniques that target specific biases like batch effect correction methods (e.g., LOESS normalization [46], SVA [47] or ComBat [48], [49]). HRMS datasets usually take up large amounts of hard drive storage (e.g., dozens of gigabytes per 100 samples), and have very high complexity (e.g., randomization, data hierarchy, time series). Therefore, data management and archiving in LC-MS, as well as other study data, is crucial for traceability of the study.

## Data analysis and Clinical translation

**Fig. 6:**
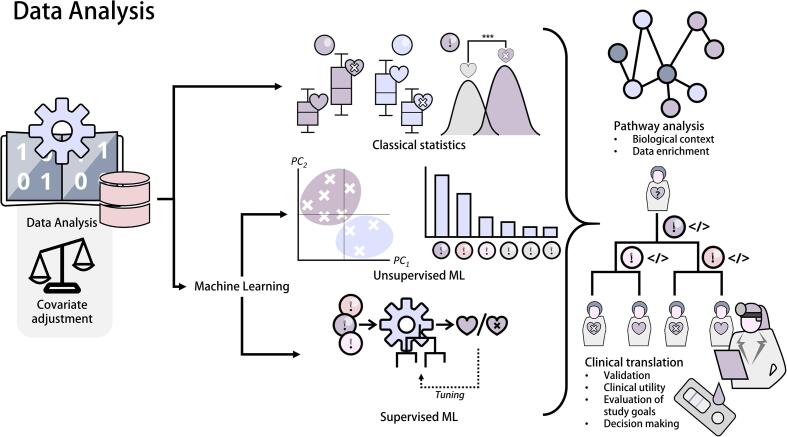
Data analysis can take place between the fields of classical statistics and machine learning, which often overlap. Statistics obtain p-values, usually in uni- or bivariate analysis, to claim significance, while machine learning approaches strive for the best possible test metrics, such as optimal prediction accuracy. Machine learning can be further divided into unsupervised and supervised approaches. Unsupervised approaches act independently of pre-defined sample groups to develop hypotheses and explore data (although inferences are also possible). In contrast, supervised models usually aim for optimal regression or classification in the context of the research scope. Therefore, both supervised models and classical statistics can be used to confirm or reject hypotheses. Pathway analyses can integrate the obtained data into a broader biological context. Finally, resulting findings and potential biomarkers or biomarker panels need to be clinically validated, demonstrate clinical benefit, and be practical in order to be translated into clinical routine.

Data analysis specific to the clinical scope of the study requires domain knowledge when pursuing specific questions. Before cohorts are evaluated, data sets may need to be adjusted for covariates (e.g., age distribution among groups) to avoid confounding [50], [51]. A mix of classical statistics and machine learning applications is employed for data investigation [Fig. 6] [52], with classical statistics focusing on explaining phenomena, while machine learning methods focus on prediction [53]. Unsupervised machine learning algorithms offer a general way of learning underlying structures of datasets; principal component analysis (PCA) and hierarchical clustering are instrumental in data exploration. Besides information on patterns within the data, they are able to offer techniques of variable selection (i.e., dimensionality reduction), which is crucial in identifying potential biomarkers from hundreds or thousands of measured analytes. Supervised machine learning algorithms can put selected variables to the test and rely on powerful regression and classification algorithms, like the Random Forest (RF) or Support Vector Machines (SVM) [54]. Model validation, such as Cross-validation, must be facilitated to assess supervised machine learning model performance and exclude model overfitting [55]. Optimization of supervised machine learning models is usually performed through a process of model parameter tuning and model assessment [54]. Pathway analysis helps to embed findings in a broader biological context and form hypotheses and make causal assumptions [56].

Besides analytical and clinical validity, biomarkers or biomarker panels need to prove their utility by being practical and broadly applicable in a clinical context. However, validating biomarkers poses methodological and general challenges. A common problem in initial biomarker research is the lack of power due to small sample sizes, which increases the risk for confounders and reduces the applicability of machine learning methods. A high number of potential biomarkers, generated from a small sample size can thus be a result of overfitting [57]. Awareness of this is of particular interest, as there is no consensus on the best way of feature selection and machine learning methods are often selected for isolating a set of variables [58]. Furthermore, metabolites are especially prone to vary due to various covariates, such as age, gender, and diet. Therefore, if feature selection steps result in a high number of analytes, special caution needs to be taken to avoid such biases (e.g., by stratifying the cohort properly). Additionally, due to the complexity of many diseases and subsequent systemic changes, individual biomarkers are unlikely to emerge for diagnostic or prognostic purposes [59]. To overcome this issue, validation calls for increasing the sample size with an independent cohort to confirm selectivity, stability, and repeatability of the measurement and raise statistical power [60]. Moreover, there needs to be an emphasis on the utility of potential biomarkers for their end users throughout the research process. Insufficient interdisciplinary exchange can obscure the lack of clinical relevance, which is not only influenced by predictive power, but also by cost-effectiveness in the clinical laboratory (in terms of instruments, work space, expertise, and staff allocation) and benefit for physicians and their patients in making therapy decisions [61]. While many biomarkers fail at this stage, those that are successful can improve clinical decision making and/or elucidate novel therapeutic targets [46], [47].

## Quality control & quality assurance

**Fig. 7:**
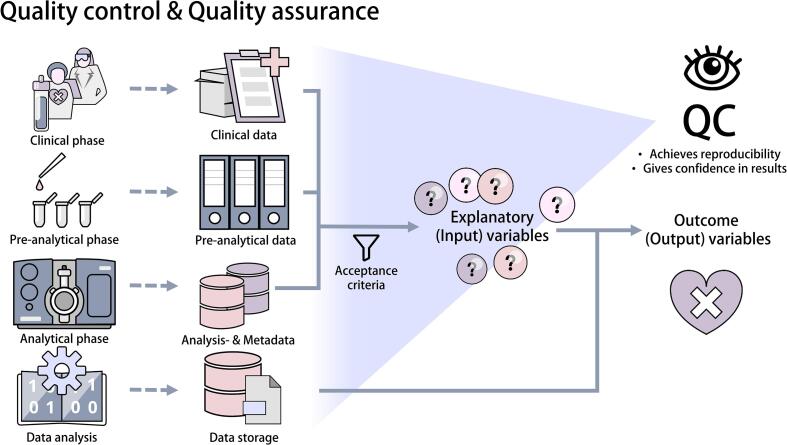
Quality control and quality assurance accompany the entire process of clinical research projects. Standard operating procedures, as well as good documentation practices, are essential for quality assurance in all stages of research. Data management of clinical data, metadata, data on (pre-)processing, analysis, and evaluation of results allows for traceability across the entire study. Clinical data, documentation on sample processing, analytical phases, and detailed reporting on the data analysis facilitate comprehension of the results and are necessary for reproducibility and validity of the study.

Published potential biomarkers found in LC-MS studies routinely fail in clinical translation, due to inadequate reporting, low quality control, low reproducibility and overfitting of models to available data sets [62]. Other issues that hinder the implementation of valuable biomarkers include biases in cohort selection and evaluation, as well as insufficient independent validation of research results. Quality management measures should be put in place to provide quality control (QC) and quality assurance (QA) throughout the entire research process, so as to improve the reliability and reproducibility of the data and experiments [Fig. 7]. This can be achieved by providing specific instructions for each step of the analytical process and requiring frequent documentation, which will enable traceability [36]. Documentation for quality control purposes should also include recording metadata that describe the study results. To enhance reproducibility across studies, national and international guidelines should be developed to harmonize quality control protocols [63].

## Funding

This work was supported by the Deutsche Forschungsgemeinschaft Sonderforschungsbereich SFB 1039/Z01 “Krankheitsrelevante Signaltransduktion durch Fettsäurederivate und Sphingolipide”, by Fraunhofer Cluster of Excellence for Immune Mediated diseases CIMD and the HIPPOCRATES project. HIPPOCRATES has received funding from the Innovative Medicines Initiative 2 Joint Undertaking (JU) under grant agreement no. 101007757. The JU receives support from the European Union’s Horizon 2020 research and innovation program and EFPIA. BB is supported by grants from the National University of Singapore via the Life Sciences Institute.

## Declaration of Competing Interest

The authors declare that they have no known competing financial interests or personal relationships that could have appeared to influence the work reported in this paper.
