# Supplementary material for: Small molecule biomarker discovery: Proposed workflow for LC-MS-based clinical research projects
Source: J Mass Spectrom Adv Clin Lab. 2023 Feb 17;28:47–55. doi: 10.1016/j.jmsacl.2023.02.003 (PMC9982001; doi:10.1016/j.jmsacl.2023.02.003)
Supplement: Supplementary Data 1 [file mmc1.pptx]

## Slide 1
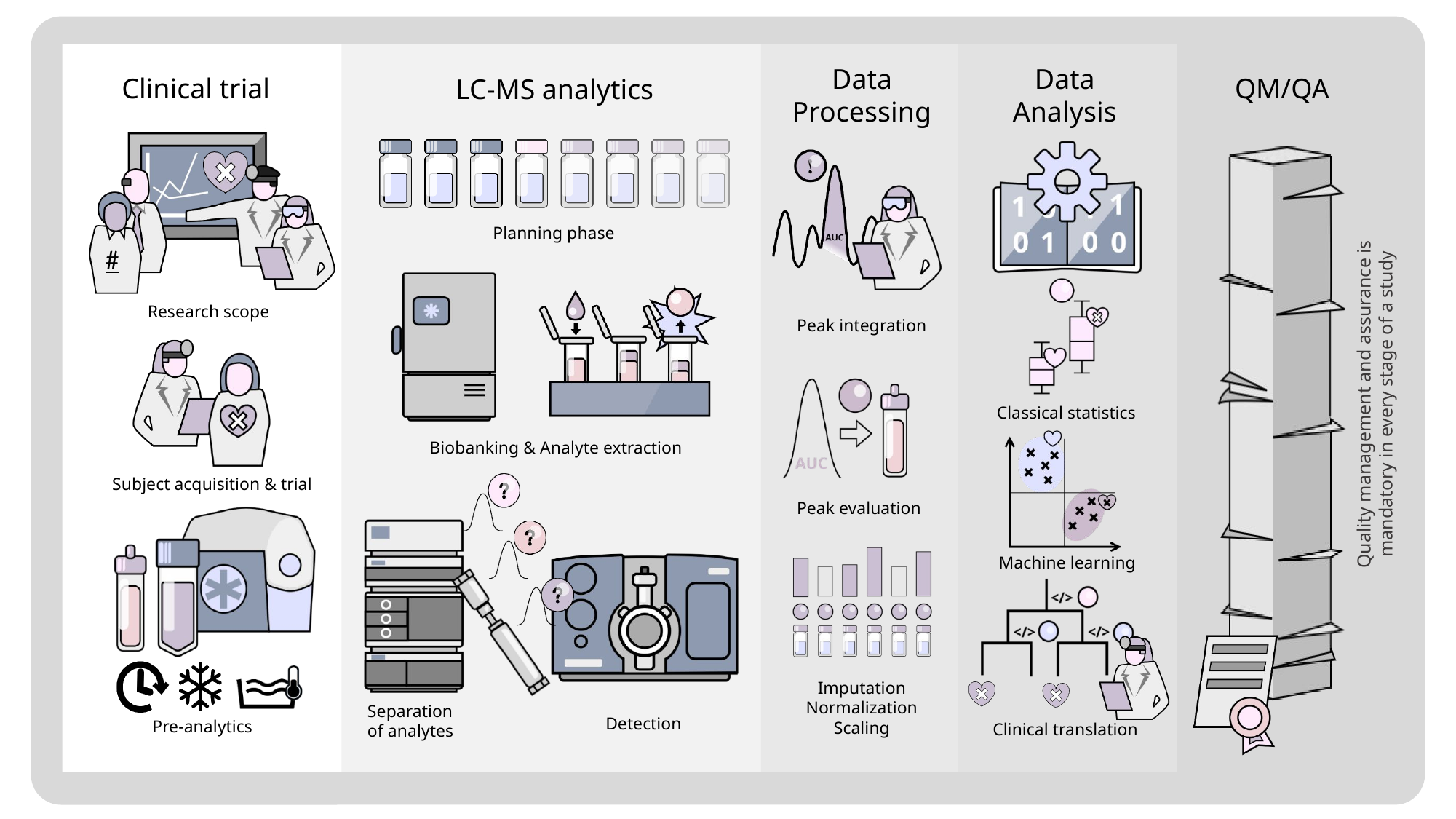

Data Analysis
Data Processing
Clinical trial
QM/QA
LC-MS analytics
#
Planning phase
Research scope
Peak integration
Quality management and assurance is mandatory in every stage of a study
Classical statistics
Machine learning
Biobanking & Analyte extraction
Separation
of analytes
Detection
Subject acquisition & trial
Peak evaluation
Imputation
Normalization
Scaling
Pre-analytics
Clinical translation

## Slide 2
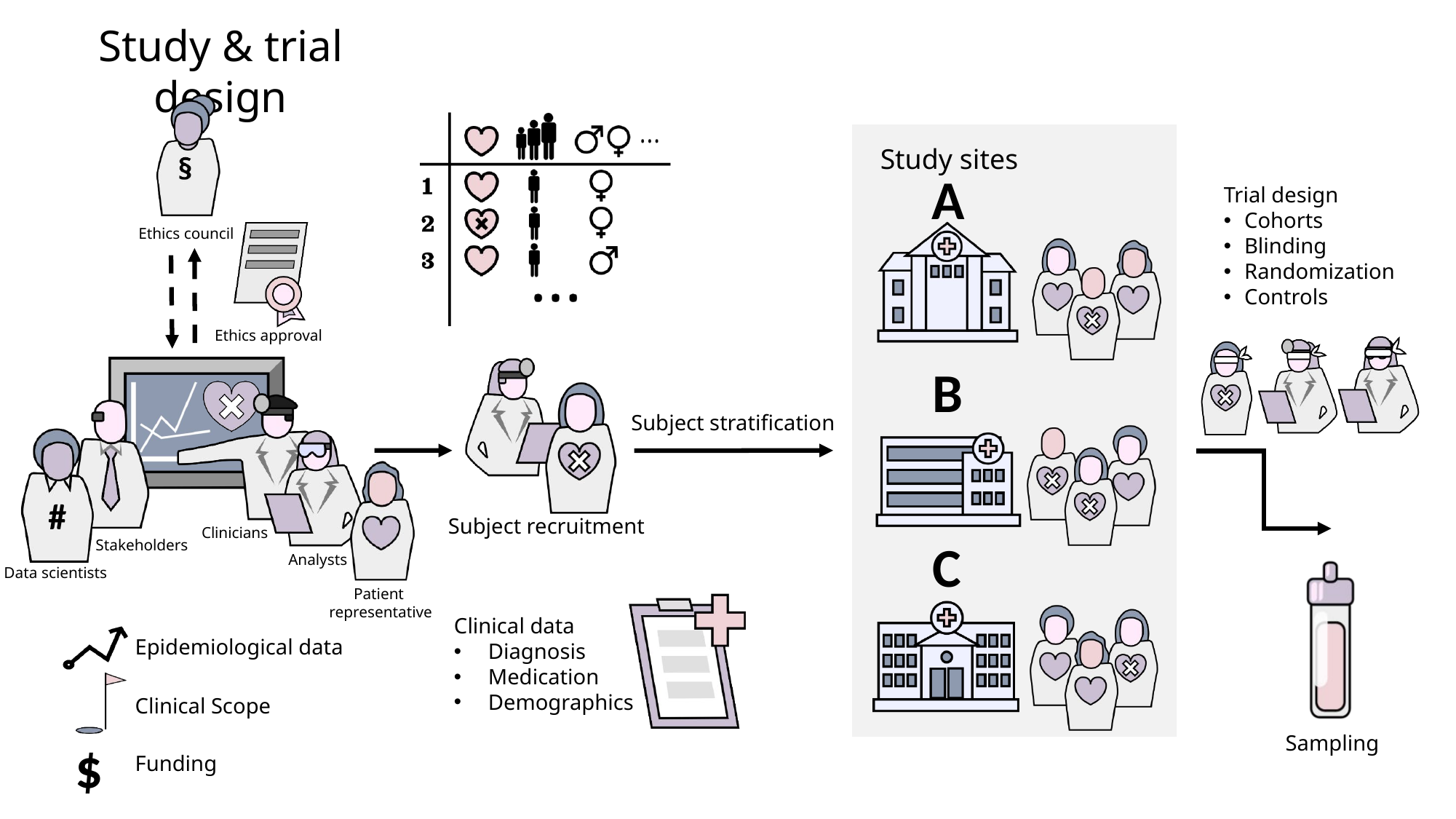

Study & trial design
Ethics council
Study sites
A
B
C
Trial design
Cohorts
Blinding
Randomization
Controls
Ethics approval
#
Clinicians
Stakeholders
Analysts
Data scientists
Patient
representative
Subject recruitment
Subject stratification
Sampling
Epidemiological data
Clinical Scope
$
Funding
Clinical data
Diagnosis
Medication
Demographics

## Slide 3
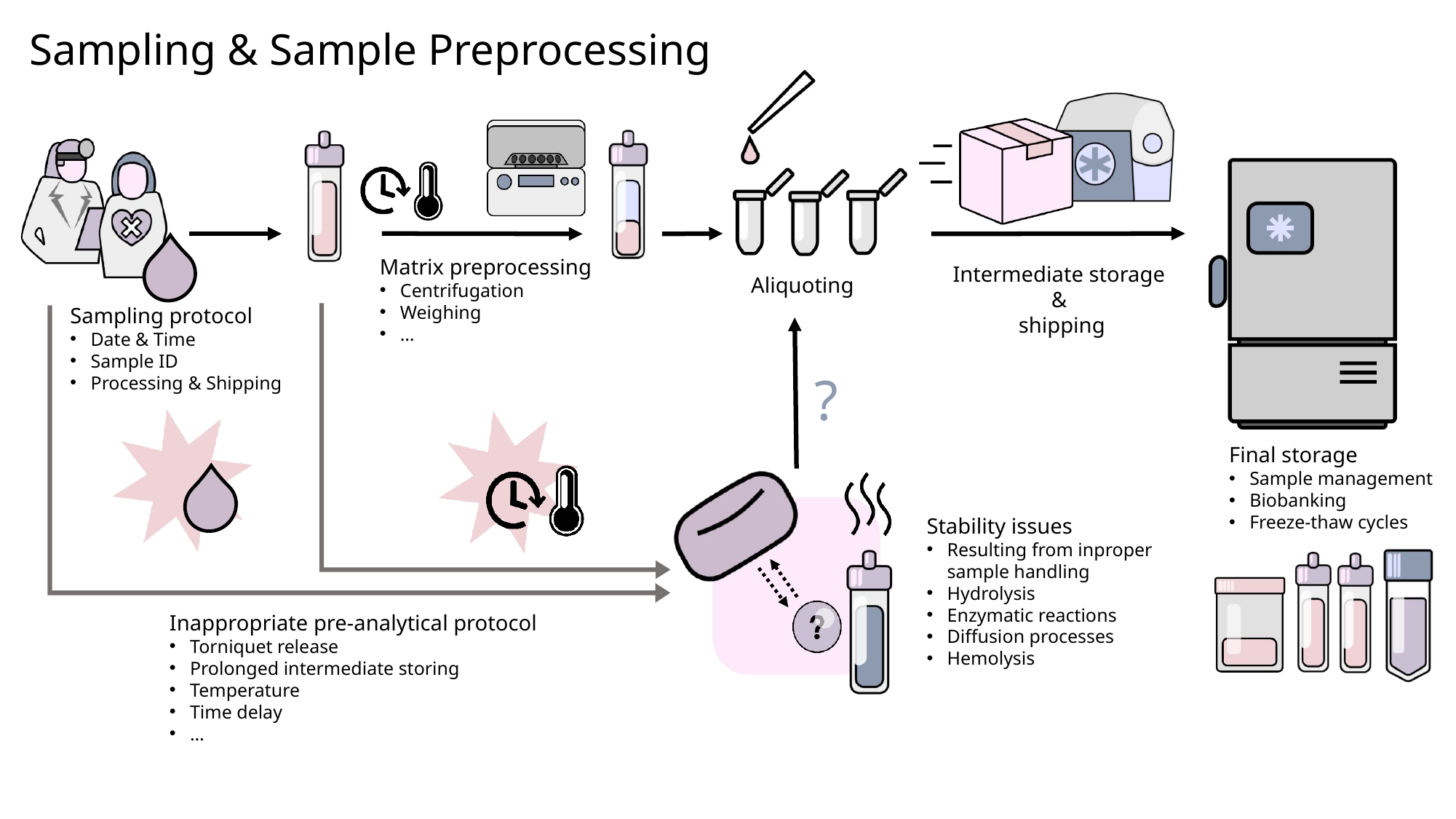

Sampling & Sample Preprocessing
Matrix preprocessing
Centrifugation
Weighing
…
Intermediate storage
&
shipping
Aliquoting
Sampling protocol
Date & Time
Sample ID
Processing & Shipping
?
Final storage
Sample management
Biobanking
Freeze-thaw cycles
Stability issues
Resulting from inproper
sample handling
Hydrolysis
Enzymatic reactions
Diffusion processes
Hemolysis
Inappropriate pre-analytical protocol
Torniquet release
Prolonged intermediate storing
Temperature
Time delay
…

## Slide 4
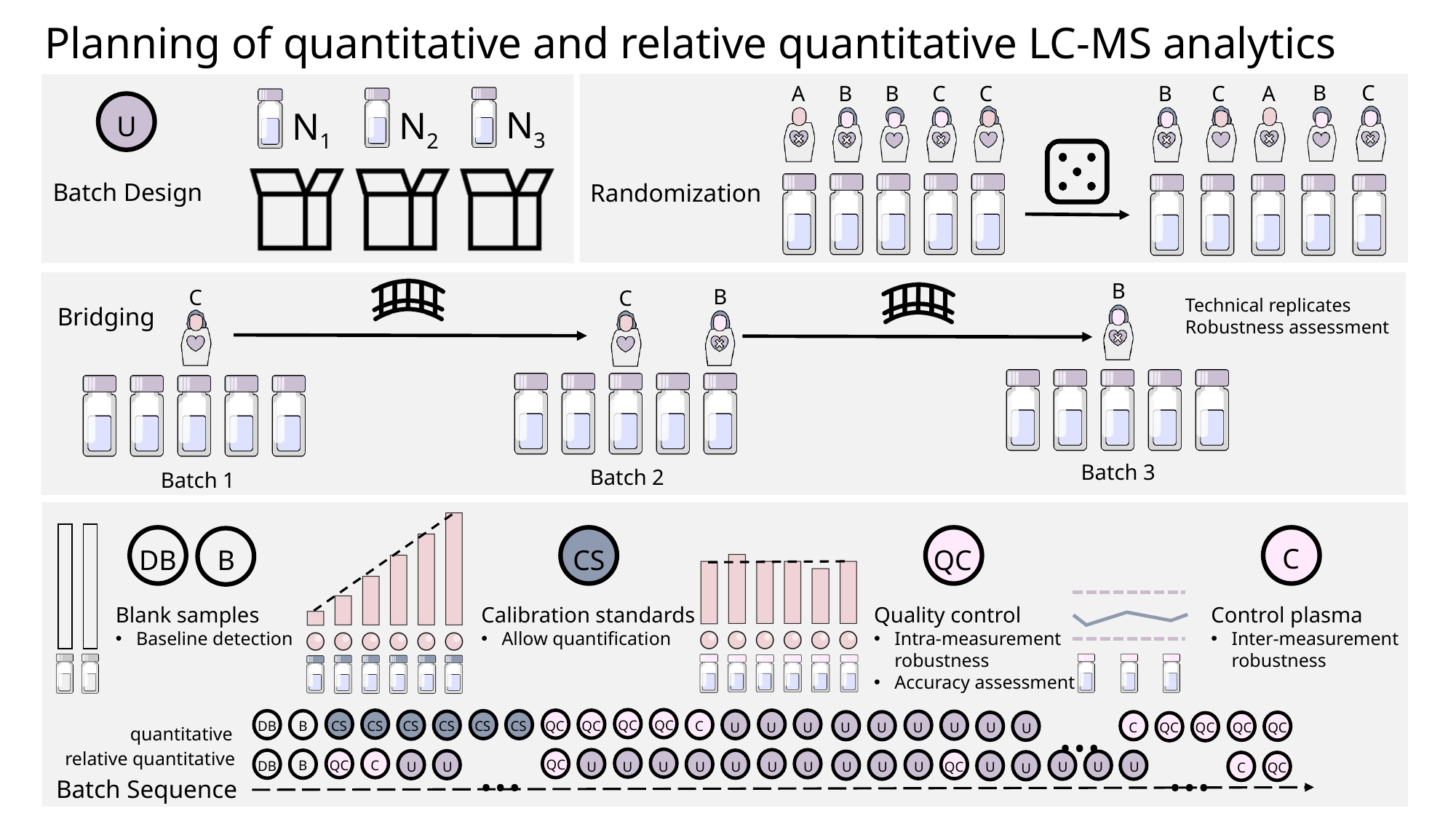

Planning of quantitative and relative quantitative LC-MS analytics
N3
N2
N1
Batch Design
C
B
C
A
B
B
C
C
B
A
Randomization
U
B
B
C
Batch 1
C
Batch 3
Batch 2
Technical replicates
Robustness assessment
Bridging
C
DB
B
CS
QC
Blank samples
Baseline detection
Calibration standards
Allow quantification
Quality control
Intra-measurement
robustness
Accuracy assessment
Control plasma
Inter-measurement robustness
…
QC
QC
QC
QC
C
DB
B
CS
CS
CS
CS
CS
CS
U
U
U
U
U
U
U
U
C
QC
QC
QC
QC
U
quantitative
…
…
relative quantitative
QC
QC
C
B
DB
U
U
U
U
U
U
U
U
U
U
U
U
QC
U
U
U
U
C
QC
U
Batch Sequence

## Slide 5
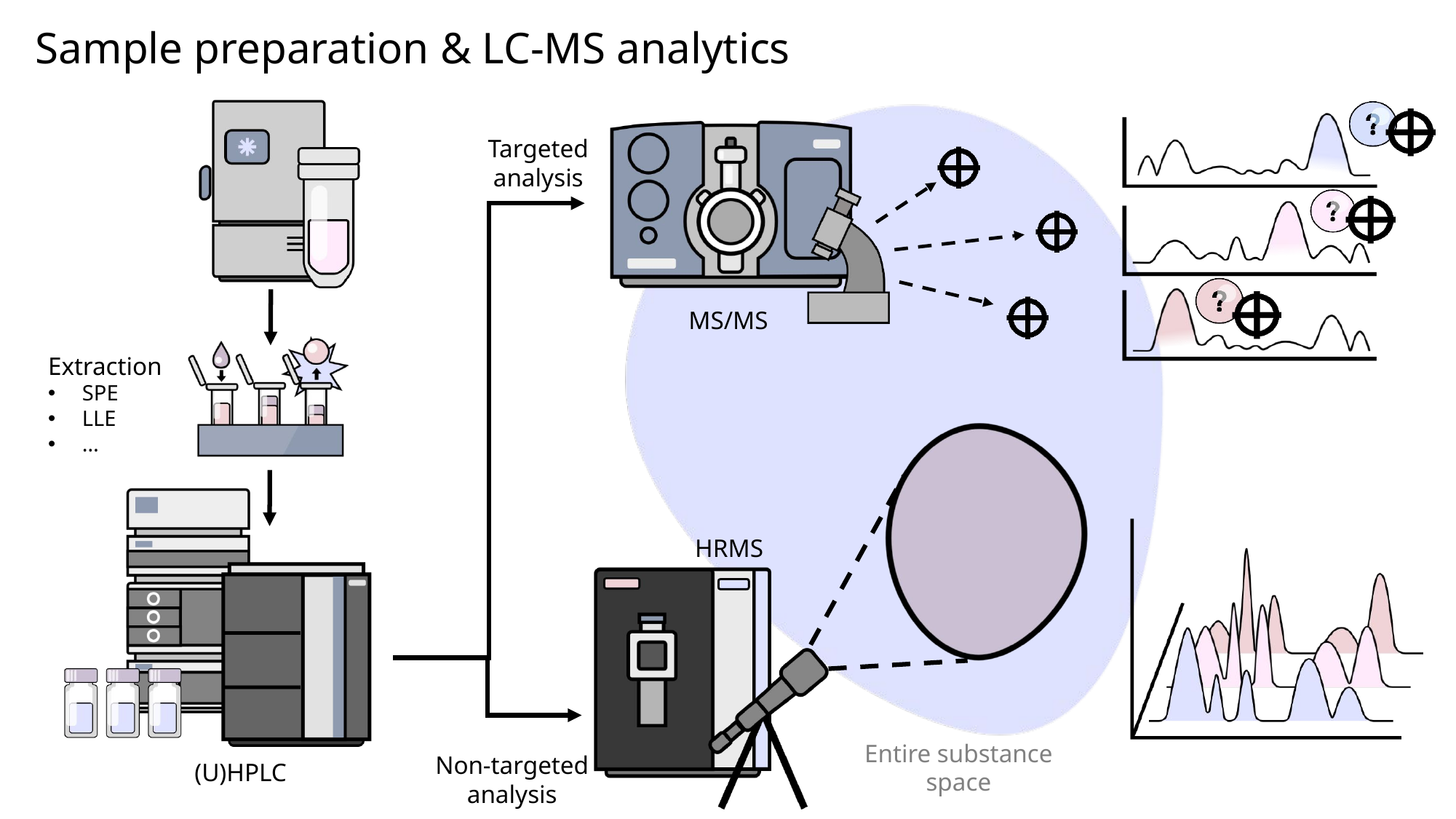

Sample preparation & LC-MS analytics
MS/MS
Targeted analysis
Extraction
SPE
LLE
…
HRMS
(U)HPLC
Entire substance space
Non-targeted
analysis

## Slide 6
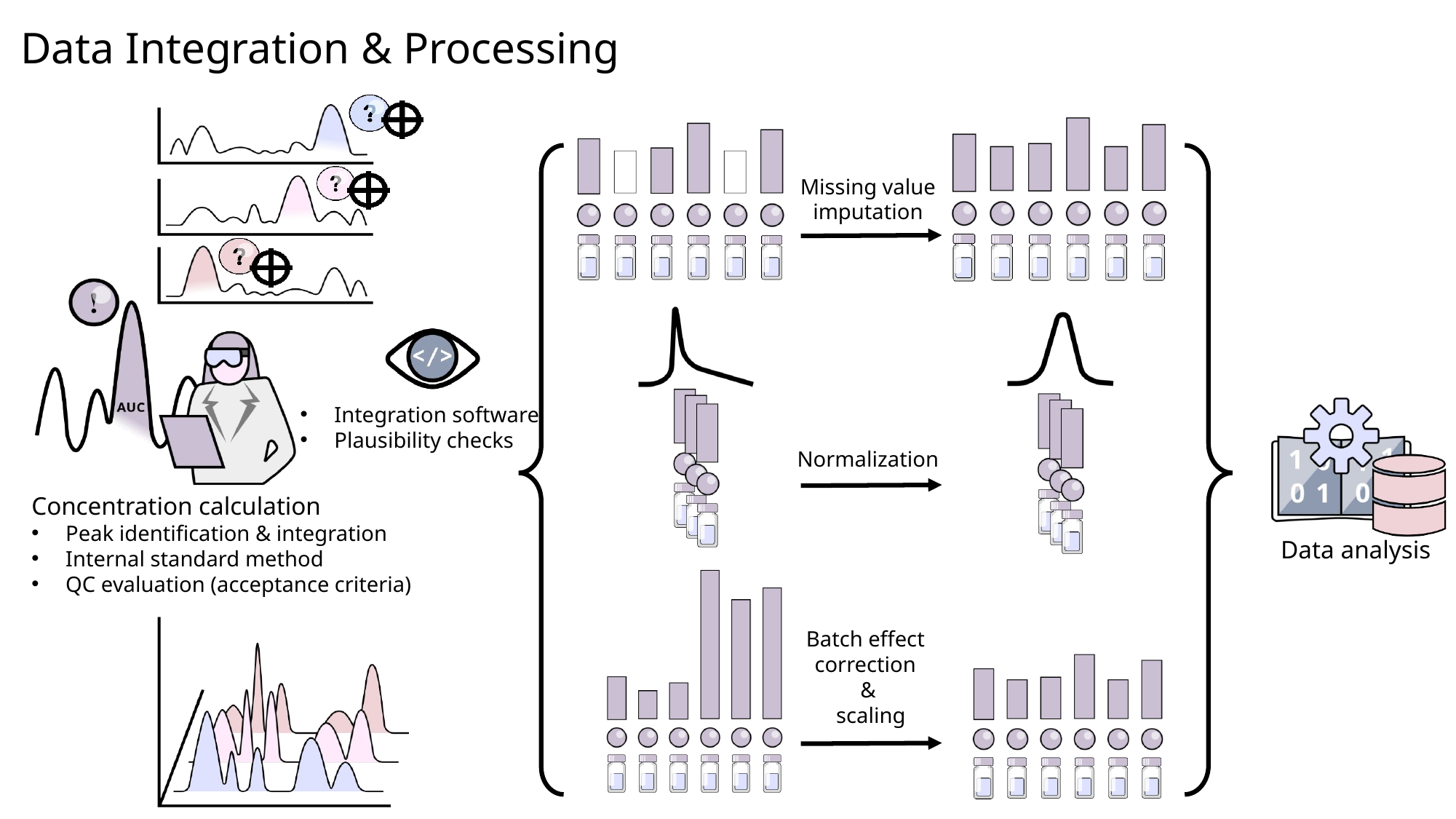

Data Integration & Processing
Missing value
imputation
Integration software
Plausibility checks
Normalization
Concentration calculation
Peak identification & integration
Internal standard method
QC evaluation (acceptance criteria)
Data analysis
Batch effect
correction
&
 scaling

## Slide 7
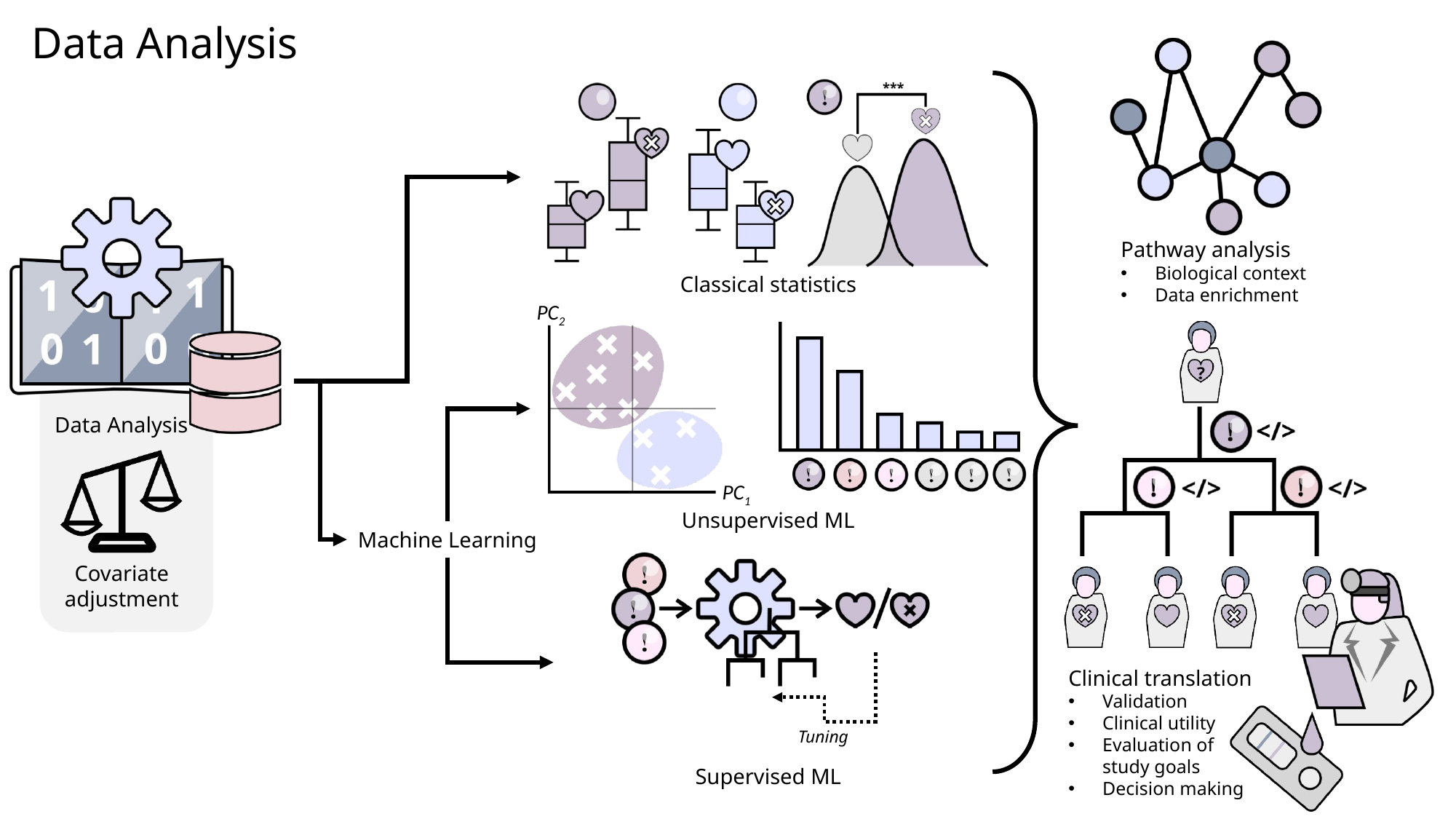

Data Analysis
Data Analysis
Covariate adjustment
Pathway analysis
Biological context
Data enrichment
Classical statistics
PC2
PC1
Unsupervised ML
Machine Learning
Clinical translation
Validation
Clinical utility
Evaluation of
study goals
Decision making
Tuning
Supervised ML

## Slide 8
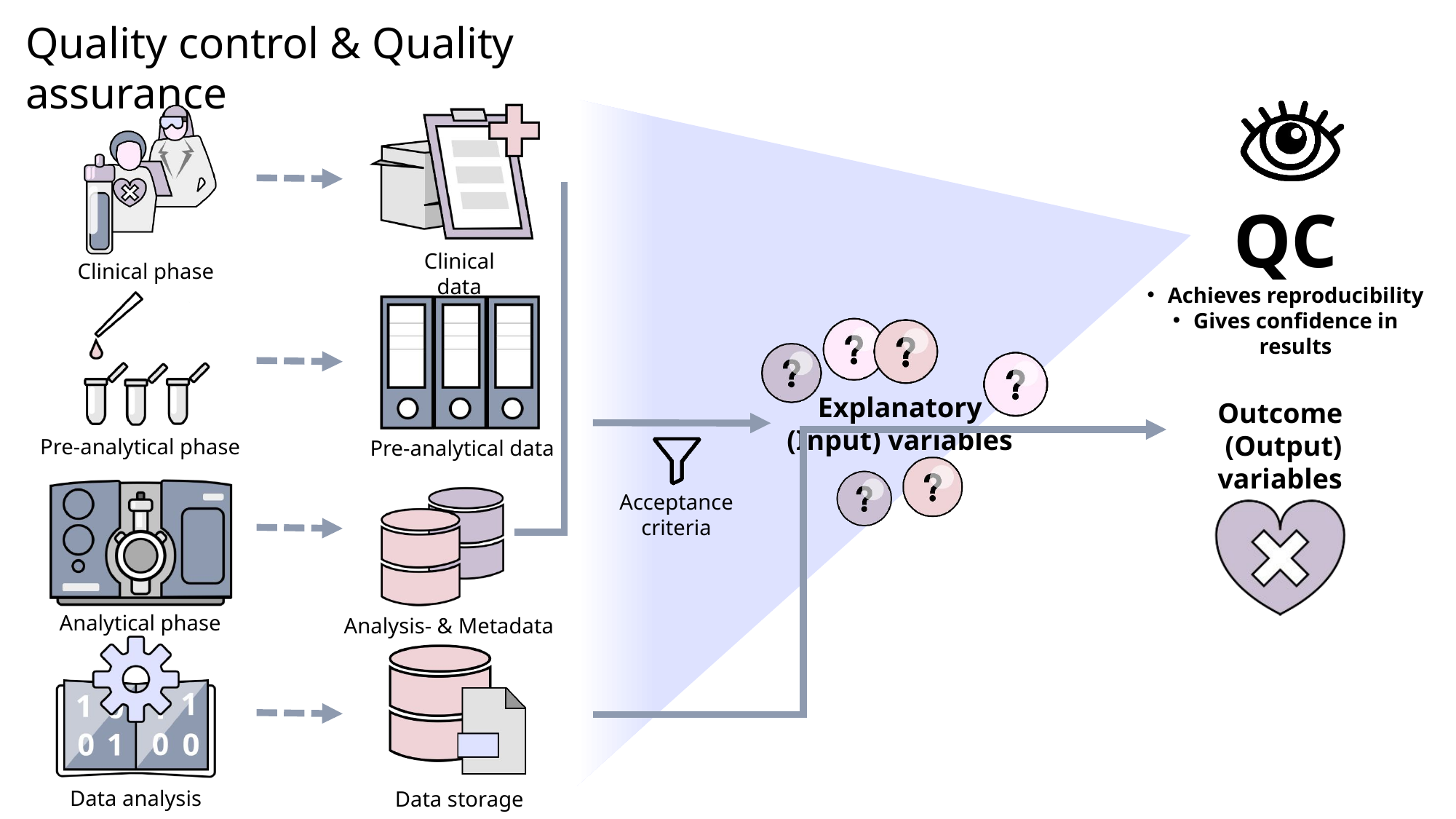

Quality control & Quality assurance
QC
Achieves reproducibility
Gives confidence in results
Clinical data
Clinical phase
Explanatory (Input) variables
Outcome
 (Output) variables
Pre-analytical phase
Pre-analytical data
Acceptance
criteria
Analytical phase
Analysis- & Metadata
Data analysis
Data storage
